# ﻿*Bertieraacutiflora* (Rubiaceae, Bertiereae), a new combination based on the overlooked *Psychotriaacutiflora*

**DOI:** 10.3897/phytokeys.251.140272

**Published:** 2025-01-17

**Authors:** Andreas Berger

**Affiliations:** 1 Department of Botany, Natural History Museum Vienna, Burgring 7, Vienna, 1010, Austria Department of Botany, Natural History Museum Vienna Vienna Austria; 2 Department of Botany and Biodiversity Research, University of Vienna, Rennweg 14, Vienna, 1030, Austria University of Vienna Vienna Austria

**Keywords:** *Bertiera*, Flora of Ecuador, new combination, *Psychotria*

## Abstract

*Psychotriaacutiflora* was described in 1830 by de Candolle in his Prodromus, and was based on a single collection by Thaddäus Haenke from near Guayaquil, Ecuador. Although the identity of the name has never been studied since its original publication, it is currently treated as a species endemic to Ecuador. It is shown here that the type of the name belongs to *Bertieraprocumbens*, which was described much later. Therefore, the new combination *Bertieraacutiflora* is provided here for the Columbian and Ecuadorian endemic and a lectotype is designated for the name.

## ﻿Introduction

*Psychotriaacutiflora* DC. (Rubiaceae) was described by Augustin-Pyramus de Candolle (1778–1841) in the fourth volume of his *Prodromus* (de Candolle 1830). The name was apparently not used until Kuntze (1891) transferred it to the genus *Uragoga* Baill. in which he included essentially all *Psychotria* L. and related genera. *Psychotriaacutiflora* was not treated in the Flora of Ecuador (Andersson and Ståhl 1999), but was provisionally accepted in the Catalogue of the Vascular Plants of Ecuador (Taylor and Andersson 1999). Probably on the basis of this catalogue, *Psychotriaacutiflora* is currently considered as a species endemic to Ecuador (POWO 2024). Apart from Taylor and Andersson (1999), who did not examine the type but suggested that the name might belong to *Bertiera*, the name has apparently been overlooked, and its identity is studied here for the first time since its original publication almost two centuries ago.

*Psychotriaacutiflora* was based on flowering material collected by the Bohemian botanist Thaddäus X. P. Haenke (1761–1816) during the Malaspina Expedition (1789–1794) in the vicinity of Guayaquil, Ecuador. During the Malaspina expedition, Haenke collected ample material, and part of it was sent to the Spanish authorities who funded the expedition. In addition, Haenke sent his private share of his collection, an estimated 15,000 specimens, to a trading company in Cádiz for safekeeping until his return. However, he remained in South America until his death and the specimens were forgotten for some time. In 1821, Kaspar M. Sternberg rediscovered the material and organized its transfer and study in Prague (Anonymous 1826), where it formed the basis of C. B. Presl’s *Reliquiae Haenkeanae* (Presl 1825–1835; see Stearn 1938).

The Rubiaceae of the expedition were initially studied by Friedrich Gottlieb Bartling (1798–1875), botanist in Göttingen and one of the contributors to Presl’s incomplete Reliquiae Haenkeanae. Due to delays in publication, selected families, including the Rubiaceae, were loaned to de Candolle for study. He included the material in his Prodromus, returned the specimens and kept some fragments for his herbarium (O. Sida, pers. comm.). On the basis of this material (“v. s. in h. Haenk.”), de Candolle described *Psychotriaacutiflora* taking up the epithet of Bartling’s unpublished name ‘Guettarda acutiflora’.

## ﻿Results and discussion

**Figure 1.**
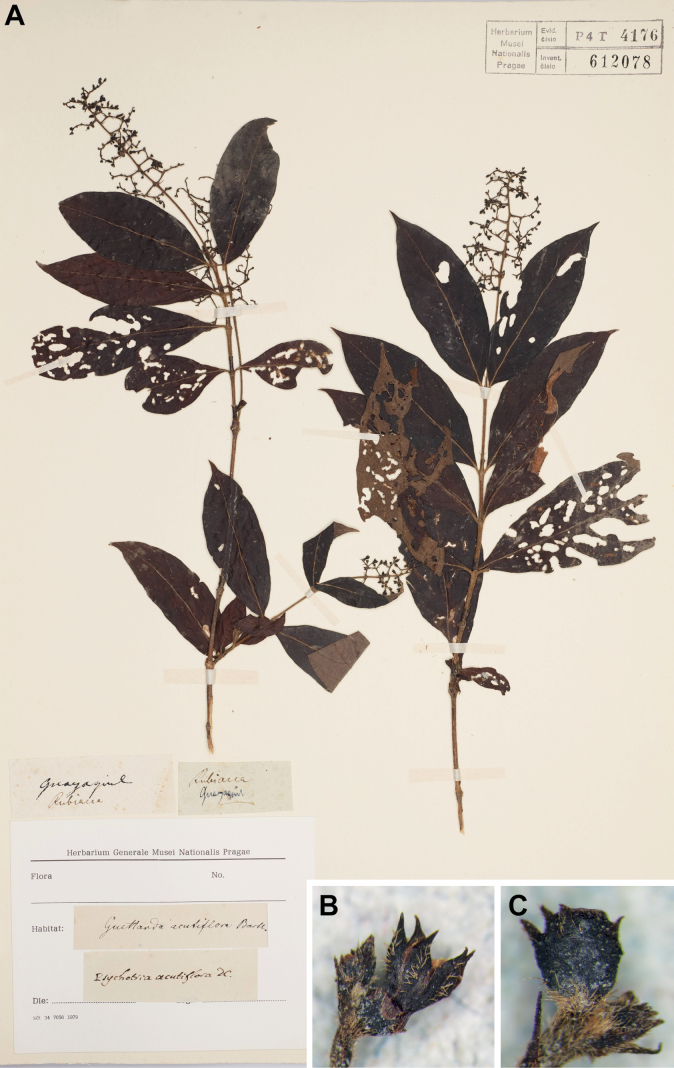
Type specimens of *Psychotriaacutiflora* DC. (= *Bertieraacutiflora*, comb. nov.) **A** lectotype in the herbarium of the National Museum in Prague (PR 612078; photo: herbarium PR) **B, C** details of isolectotype in the Prodromus herbarium, Conservatoire et Jardin botaniques de Genève (G-DC, G00667379) **B** flower **C** young fruit (photos: A. Berger).

In the present study, the type fragment in the Prodromus Herbarium of de Candolle (G-DC) at the Conservatoire et Jardin botaniques de Genève was examined. The specimen has two pairs of leaves and an inflorescence in the late flowering stage with a few flower buds and immature fruits, and most of the flowers with the corolla already fallen off. In addition, a complete specimen with two flowering branches was located in the herbarium of the National Museum in Prague (PR), where the majority of Haenke’s collections are kept, and both are annotated in de Candolle’s hand as *Psychotriaacutiflora* (see Fig. 1A).

The absence of raphides, the abundant pubescence of the vegetative parts, the persistent triangular stipules, the terminal, thyrsiform, bracteate inflorescences with the secondary axes dichasial at the first node and monochasial and secund at subsequent nodes, and the apiculate corolla tips immediately exclude *Psychotria* L. and suggest the genus *Bertiera* Aubl. within Bertiereae Bridson. The monogeneric tribe belongs to the predominantly paleotropic Coffeeae alliance and is sister to the paleotropic Coffeeae DC. (Kainulainen et al. 2013, 2017; Amenu et al. 2022; Kiehn and Berger 2023; Razafimandimbison and Rydin 2024). It differs from this tribe in having terminal (as opposed to paired axillary) inflorescences and (usually) numerous (as usually opposed to few) ovules per locule (Davis et al. 2007; Kainulainen et al. 2013). *Bertiera* is pantropical, and includes more than 50 species, most of which occur in tropical Africa (Robbrecht et al. 1993; Wittle and Davis 2010).

Five species of *Bertiera* are currently known from Ecuador, where the type of *Psychotriaacutiflora* was collected. All but one generally occur at lower elevations, usually below 1000 m (Andersson and Ståhl 1999; Andersson and Persson 2001). The flora of Ecuador treatment (Andersson and Ståhl 1999) recognizes four species, *Bertieraangustifolia* Benth., *Bertierabracteosa* (Donn. Sm.) B.Ståhl & L.Andersson, *Bertieraguianensis* Aubl. and *Bertieraprocumbens* K.Schum. & K.Krause. Later, another species, the montane *Bertierarugosa* L. Andersson & C.H.Perss. was described (Andersson and Persson 2001). It is only known from the type collected at about 2000 m, and is probably identical to *Bertieraviburnoides* (Standl.) J.H.Kirkbr. (Taylor 2024).

In Ecuador, *Bertieraprocumbens* is the only species that occurs predominantly west of the Andes, and the only species known from around Guayaquil and the entire Guayas province where the type of *Psychotriaacutiflora* was collected. *Bertieraprocumbens* is easily distinguished from its congeners by a number of characters: short pedicellate (vs. sessile) flowers, smooth (vs. ribbed) fruits and (2–)4-locular (vs. 5–19-locular) fruits (e.g. Andersson and Ståhl 1999; Andersson and Persson 2001). The type of *Psychotriaacutiflora* has short but clearly pedicellate flowers (see Fig. 1A, B) and de Candolle (1830) reports that the fruits are 2-locular. Thus, based on distribution and morphology, the material can be clearly identified as *Bertieraprocumbens*. Finally, the fruits are smooth which also fits this species, but they are probably too young to show this character beyond doubt (see Fig. 1C).

*Psychotriaacutiflora* has priority over *Bertieraprocumbens*, described much later by Schumann and Krause (Krause 1908) from the same region, and the name is here transferred to *Bertiera*. In addition, the complete specimen preserved in the herbarium of the National Museum in Prague (PR) is selected as the lectotype of the name (see Fig. 1A). *Bertieraprocumbens* itself was described based on a collection by H.F.A. von Eggers from Balao near Guayaquil. Eggers materials are usually found in many herbaria, but comparably few type specimens of *Bertieraprocumbens* are currently known and none bear an original annotation with the name. The duplicate in BR is a complete specimen with several branches with flowers and fruits, bears an original label from the Berlin herbarium where both K. Schumann and K. Krause worked, and is designated here as the lectotype of the name.

### ﻿Taxonomic treatment

#### ﻿ Bertiera acutiflora

Taxon classificationPlantaeGentianalesRubiaceae

(DC.) A.C.Berger comb. nov.

3D318843-F054-5B76-BB92-45229C5C4788

urn:lsid:ipni.org:names:77355304-1

Fig. 1A–C

= Bertieraprocumbens K.Schum. & K.Krause, Bot. Jahrb. Syst. 40(3): 328. 1908, syn. nov. Type: Ecuador. Guayas, Balao: “prope Bulao in silvis”, January 1892, *H. F. A. von Eggers 14282* (B†; lectotype, here designated: BR0000005305544 image!; isolectotypes: US00138193 image!, F0068504F fragm. image!, M0187082 image!, M0187083 image!).

##### Basionym.

*Psychotriaacutiflora* DC., Prodr. 4: 506. 1830. ≡ *Uragogaacutiflora* (DC.) Kuntze, Revis. Gen. Pl. 2: 959. 1891. Type: Ecuador. Guayas, Guayaquil: “Guayaquil”, 1790, *T. Haenke s.n.* (lectotype, here designated: PR 612078 image!; isolectotype: G-DC 00667379!).

##### Distribution.

Ecuador: Cañar (*J. H. Vargas López et al. 512*, MO-1562740 image!), Chimborazo, Esmeraldas, Guayas, Los Ríos, Manabí, Pichincha. Colombia: Island Gorgona (Andersson and Ståhl 1999).

## Supplementary Material

XML Treatment for Bertiera acutiflora

## ﻿Additional information

### Author ORCIDs

Andreas Berger https://orcid.org/0000-0003-3902-3827

### Data availability

All of the data that support the findings of this study are available in the main text.
